# Plasmid DNA immunization with *Trypanosoma cruzi* genes induces cardiac and clinical protection against Chagas disease in the canine model

**DOI:** 10.1186/1297-9716-43-79

**Published:** 2012-11-13

**Authors:** Olivia Rodríguez-Morales, M Magdalena Pérez-Leyva, Martha A Ballinas-Verdugo, Silvia C Carrillo-Sánchez, J Luis Rosales-Encina, Ricardo Alejandre-Aguilar, Pedro A Reyes, Minerva Arce-Fonseca

**Affiliations:** 1 Department of Molecular Biology, Instituto Nacional de Cardiología “Ignacio Chávez”, Juan Badiano No. 1, Col. Sección XVI, Tlalpan, Mexico City, 14080, Mexico; 2 Department of Infectomics and Molecular Pathogenesis, Centro de Investigación y de Estudios Avanzados del I.P.N., Av. Instituto Politécnico Nacional No. 2508, Col. San Pedro Zacatenco, Gustavo A. Madero, Mexico City, 07360, Mexico; 3 Department of Parasitology, Escuela Nacional de Ciencias Biológicas del I.P.N., Prolongación de Carpio y Plan de Ayala, Col. Sto. Tomás, Miguel Hidalgo, Mexico City, 11340, Mexico; 4 Direction of Research, Instituto Nacional de Cardiología “Ignacio Chávez”, Juan Badiano No. 1, Col. Sección XVI, Tlalpan, Mexico City, 14080, Mexico

## Abstract

The only existing preventive measure against American trypanosomosis, or Chagas disease, is the control of the transmitting insect, which has only been effective in a few South American regions. Currently, there is no vaccine available to prevent this disease. Here, we present the clinical and cardiac levels of protection induced by expression to *Trypanosoma cruzi* genes encoding the *Tc*SP and *Tc*SSP4 proteins in the canine model. Physical examination, diagnostic chagasic serology, and serial electrocardiograms were performed before and after immunization, as well as after experimental infection. We found that immunization with recombinant plasmids prevented hyperthermia in the acute phase of experimental infection and produced lymphadenomegaly as an immunological response against the parasite and additionally prevented heart rate elevation (tachycardia) in the acute and/or chronic stages of infection. Immunization with *T. cruzi* genes encoding the *Tc*SP and *Tc*SSP4 antigens diminished the quality and quantity of the electrocardiographic abnormalities, thereby avoiding progression to more severe developments such as right bundle branch block or ventricular premature complexes in a greater number of dogs.

## Introduction

Chagas disease (CD) is endemic to the American continent, and 25 million people are at risk in Latin America [1]. There are several *T. cruzi* reservoirs; the dog is the most important domestic species in the infective cycle, mainly because the dog serves as a considerable source of nourishment for the triatomine insects, and the fact that the dogs eat infected bugs, thus increasing the risk of transmission within human dwellings [2]. It is estimated that there are 3500 new cases per year in Mexico [3], and the dog population seroprevalence is between 1.6 and 21%, depending on the geographical region of the country [4-7]. Therefore, there is a significant number of dogs that could be acting as reservoirs, thus creating a public health problem.

Currently, a major stumbling block for research efforts striving to elucidate the mechanisms of acute and chronic CD pathogenesis is the lack of a suitable animal model. It has been shown that dogs develop diffuse chronic myocarditis with histological and electrocardiographic changes that are also found in humans [8-12]; therefore, this animal represents a useful experimental model that is gaining attention in the CD research field. The clinical signs in Chagas-infected dogs during both the chronic and acute stages closely resemble the symptoms of human disease [13-15].

In spite of the success of vaccines, there are still numerous pathogens, and in particular protozoan parasites, against which there are still no effective vaccine. Specifically, vaccine development against CD has been dramatically limited because of an extensive debate on the mechanisms involved in disease pathology. However, the discovery that the direct injection of plasmid DNA encoding foreign proteins could lead to endogenous protein biosynthesis and a specific immune response against it has resulted in new directions for vaccine development, particularly if the vaccine is capable of inducing a Th1 immune response and the activation of cytotoxic CD8+T cells [16].

In a previous study that used a murine model, plasmid pBCSSP4 and pBCSP immunizations, which contained the genes encoding the *Trypanosoma cruzi Tc*SSP4 and *Tc*SP proteins, respectively, were found to be effective at inducing antibodies that were augmented after the boost. Protection during the acute phase of the disease was partial because there was a 64% reduction in parasitemia with plasmid pBCSSP4 and a 72.9% reduction with pBCSP injection compared to controls. Survival was 75% in pBCSSP4-immunized mice and 100% in those immunized with pBCSP. This study demonstrated protection during the chronic phase of the disease because histological analysis showed that the immunization diminished cardiac damage [17,18].

In this study, the canine model was used to determine the level of clinical protection given by immunization with pBCSSP4 and pBCSP plasmids to protect against experimental CD in dogs. A general physical examination was performed for each subject with special regards for body temperature, exploration of lymph nodes, and heart rate; electrocardiographic recordings were performed to evaluate cardiac electrical conduction abnormalities during the acute and chronic stages.

## Materials and methods

### Animals

Thirty male and female Beagle dogs, aged 5 (± 1) months and weighing 8.52 (± 1.55) kg, were used; all animals had been immunized against the main canine diseases, and had received intestinal deworming. A standardized enzyme-linked immunosorbent assay (ELISA) [19] was used to diagnose CD in the dogs to rule out any previous natural infections. Animal handling followed the established guidelines of the International Guiding Principles for Biomedical Research involving Animals and the Norma Oficial Mexicana [20] Technical Specifications for the Care and Use of Laboratory Animals, and the experimental protocol was approved by the Bioethics Committee of the Instituto Nacional de Cardiología, Ignacio Chávez.

### Plasmid DNA construction, purification and immunization

The gene encoding *Tc*SSP4 (amastigote-specific protein) was obtained from the pBluescriptSk-Am230 plasmid [21] as a 2.2 kb *Eco* RI (Boehringer Mannheim) fragment. The gene encoding *Tc*SP (*trans*-sialidase protein) was released from pBluescriptSk-A83 plasmid (Rosales-Encina JL, unpublished observations) as a 6.0 kb *Eco* RI fragment. The complete open reading frames of the *T. cruzi TcSSP4* and *TcSP* genes were inserted into the multiple cloning site of the commercially available eukaryotic expression vector pBK-CMV (Stratagene) to generate 2 constructs, pBCSSP4 and pBCSP, respectively. The genes were placed under the control of the cytomegalovirus promoter and were inserted downstream of a Kozak consensus sequence in-frame with an initiation codon. Colonies containing the plasmids with the insert in the correct orientation were selected. The plasmids were grown in XL1-Blue *E. coli* and purified using the QIAGEN Plasmid Mega Kit in accordance with the manufacturer's instructions. The expression of *Tc*SSP4 and *Tc*SP was analyzed using HeLa cells in vitro prior to an immunization trial of the dogs. The recombinant plasmids or control empty plasmid were transfected to HeLa cells using a transfection reagent (FuGENE 6, Boehringer Mannheim) in accordance with the protocol provided by the manufacturer. The expression of *Tc*SSP4 and *Tc*SP was analyzed by Western blotting using an anti-SSP4 or anti-SP polyclonal antibody as a primary antibody 2 days after transfection. Five experimental groups with 6 dogs each were made, and the following were infected 15 days after the corresponding treatment: 1) pBCSP plasmid-immunized, 2) pBCSSP4 plasmid-immunized, 3) pBK-CMV empty cloning vector-immunized, and 4) mock-immunized with physiologic saline solution (SS). The fifth group corresponding to the control was not subjected to any experimental procedure. The dogs were immunized with DNA twice at 15-day intervals by intramuscular injection between the semitendinosus and semimembranosus muscles of the pelvic limbs using a 3-mL syringe with a 21 G × 32 mm needle. Each single dose consisted of 500 μg DNA dissolved in 500 μL SS.

### Experimental inoculation and confirmation of *T. cruzi* infection

A well-characterized Mexican *T. cruzi* Ninoa strain maintained by serial passage in reduviid vectors was employed [22]. The infection was performed 2 weeks after the last immunization by intraperitoneal injection with 5 × 10^4^ metacyclic trypomastigotes per animal, which were obtained from urine and feces of triatomines and resuspended in SS. To confirm experimental *T. cruzi* infection in all groups, parasitemia was determined microscopically by examining freshly isolated blood samples collected from the brachiocephalic vein every third day, starting on day 10 post-infection until day 65 post-infection. Standardized ELISA and indirect immunofluorescence (IIF) were performed using methods described previously [19] to confirm experimental *T. cruzi* infection 2 months after challenge.

### Body temperature, peripheral lymph nodes, and heart rate determinations

General physical examinations were performed. For body temperature evaluation, a digital rectal thermometer (Microlife®) was used, and all peripheral lymph nodes (popliteal, submaxilary, prescapular, axillary and inguinal) were palpated and scored by a single person (MMPL) who was blinded to the experimental groups. Body temperature and peripheral lymph node determinations were registered 4 times each according to the following chronology of the experimental scheme: prior to the start of immunization (t1), before infection (t2), at the acute phase of CD (t3) and at the chronic phase of CD (t4), while heart rates were determined at t1, t3 and t4.

### Electrocardiography

Prior to the start of immunization (t1) and during the acute (t3) and chronic stages (t4) of the disease, electrocardiographic recordings were performed. Three bipolar standard leads (I, II and III), 3 augmented unipolar limb leads (aVR, aVL, and aVF), and 4 unipolar precordial thoracic leads (CV_5_RL, CV_6_LL, CV_6_LU and V_10_) were recorded. The dogs were held by an attendant in right lateral recumbency, and no chemical restraint was employed. For each tracing, the voltage was standardized at 1 mV/cm, and a paper speed of 50 mm/s was used.

### Statistical analysis

Continuous variables, such as body temperature and heart rate, were analyzed using the two-way ANOVA statistical test, establishing a correlation between each treatment group and the control group. The lymph node palpation was analyzed with a Kruskal-Wallis test.

## Results

### HeLa cell transfection

**Figure 1:**
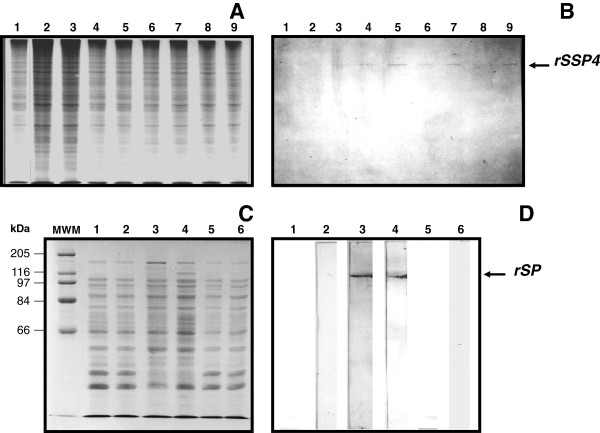
**rSSP4 and rSP protein expression in the HeLa plasmid transfected cells.** (**A**) SDS-PAGE and (**B**) western blot analysis of the pBCSSP4 plasmid transfected cell extracts; samples were taken at different time points; Lanes: 2 and 3, 8 h; 4 and 5, 24 h; 6 and 7, 48 h; 8 and 9, 56 h. The membrane was probed with the anti-*MBP::SSP4* antibody. (**C**) SDS-PAGE and (**D**) western blot analysis of the pBCSP plasmid transfected cell extracts; samples were taken at different time points; Lanes: MWM, molecular weight marker; 2, 8 h; 3, 24 h; 4, 48 h; 5, 56 h, and 6, 72 h. The membrane was probed with the anti-epimastigote whole extract antibody. Lanes 1 in (**A**), (**B**), (**C**), and (**D**) are the samples corresponding to control empty vector pBK-CMV HeLa transfected cells.

*Tc*SSP4 protein was present in the pBCSSP4 plasmid HeLa transfected cells as early as 8 h and up to 56 h post transfection, with an optimal expression level between 24 and 48 h (Figures 1a and 1b). The *Tc*SP protein expression in the eukaryotic system was determined by immunoblotting, detecting its presence only at 24 and 48 h post transfection with plasmid pBCSP (Figures 1c, and 1d). These results demonstrate that the *TcSSP4* and *TcSP* genes were correctly subcloned into the pBK-CMV vector and that the recombinant plasmids are capable of expression in eukaryotic cells.

### Diagnosis of CD in dogs

All of the experimental animals were negative for the chagasic serology test at t1 and were also in excellent health, which confirmed the absence of CD before any manipulation. Parasitemia was too low to be quantified, and parasites were only seen by direct observation after infection. The limit of detection was 200 to 400 parasites/mL of blood sample intermittently throughout all analysis. All infected/unimmunized dogs exhibited parasitemia starting on day 22 and lasting until day 55 post-infection, whereas the parasitemia of immunized/infected dogs occurred over a shorter time period, from day 32 to 46 post-infection (Table 1). At two months post-inoculation, the infection of *T. cruzi* was diagnosed by the ELISA method and was confirmed by IIF in all infected/unimmunized dogs (data not shown).

**Table 1:** **Parasitemia detection in DNA-immunized dogs with experimental *T. cruzi *infection**

**Group**	**Dog**	**Days after inoculation**									
		**10-15**	**16-20**	**21-25**	**26-30**	**31-35**	**36-40**	**41-45**	**46-50**	**51-60**	**61-65**
**pBCSP**	1							+	+		
	2					+					
	3										
	4										
	5										
	6										
**pBCSSP4**	1						+				
	2						+				
	3										
	4										
	5										
	6										
**pBK-CMV**	1					+	+				
	2					+	+	+			
	3					+	+				
	4										
	5										
	6										
**SS mock-immunized**	1						+ +	+ +		+	
	2					+	+ +				
	3						+ +	+ +	+	+	
	4						+ +			+	
	5			+ +	+ +		+ +				
	6			+	+ +						

+ Detection of 1 or 2 parasites per blood sample of fresh drop examination.

### Effect of DNA immunization on body temperature

**Figure 2:**
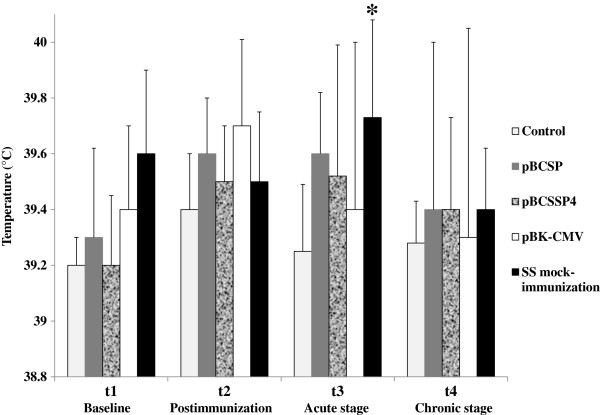
**Body temperature of DNA-immunized dogs with experimental *T. cruzi *infection.** Rectal temperature recordings from each experimental group (plasmid DNA immunized- and SS mock-immunized dogs) were compared with those of the control group (uninfected healthy animals). The values are the average of the temperature recordings ± S.D. Two-way ANOVA statistical test (* *P* ≤ 0.05).

During *T. cruzi* Ninoa strain infection, the characteristic acute stage was demonstrated by an elevation in body temperature in the SS mock-immunized and infected group. The recombinant plasmids in immunized animals avoided the temperature increase, thus demonstrating a protective effect of the plasmid DNA by eliminating this clinical sign in the acute stage of CD (Figure 2).

### Effect of experimental immunization on lymph nodes

Lymphadenomegaly in the group immunized with pBCSP plasmid was present in 50% of the animals at t2, increasing to 67% during t3 (Table 2). Approximately 50% of the pBCSSP4 immunized/infected dogs had lymph node enlargement at t2; however, all dogs showed significant lymphadenomegaly during the acute stage of the disease (t3, Table 2). This suggests that the lymphadenomegaly could be a symptom of both the infection and the reaction of vaccine, being that lymph nodes are major sites of antigen capture, detection, and initial responses during an adaptative immune response.

**Table 2:** **Lymph node palpation scores from DNA-immunized dogs with experimental *T. cruzi *infection**

**Group**	**Dog**	**Lymph node inflammation (score)**^**1**^**and percentage of dogs from each group with lymphadenomegaly**					
		**Basal t1**	**%**	**Postimmunization t2**	**%**	**Acute stage t3**	**%**
pBCSP	1	0	0	0	50	0	67
	2	0		0		0	
	3	0		0		1	
	4	0		2		1	
	5	0		1		1	
	6	0		1		2	
pBCSSP4	1	0	0	0	50	1	100*
	2	0		1		2	
	3	0		2		1	
	4	0		1		1	
	5	0		0		2	
	6	0		0		2	
pBK-CMV	1	0	0	0	17	0	83*
	2	0		0		1	
	3	0		0		1	
	4	0		0		2	
	5	0		1		1	
	6	0		0		1	
SS mock-immunized	1	0	0	0	0	1	100*
	2	0		0		1	
	3	0		0		1	
	4	0		0		1	
	5	0		0		1	
	6	0		0		1	

^1^The severity of inflammation in lymph nodes was scored on a scale from 0 to 2. A score of 0 indicated normal; 1, slightly swollen; and 2, very enlarged (scores of 1 and 2 were considered to be indicative of lymphadenomegaly). baseline = t_1_, prior to the start of the immunization, Postimmunization = t_2,_ before the infection at 15 days post-immunization, and Acute stage = t_3_, at 30 days post-infection. Popliteal, submaxillary, axillary and inguinal lymph nodes were palpated and means were compared with the Kruskal-Wallis test. Differences were considered as significant when *P* ≤ 0.05.

The lymph node enlargement after the plasmid pBK-CMV immunization was noticeable in 17% of the dogs at t2, and in the acute stage of *T. cruzi* infection (t3) the number of animals with lymph node enlargement notably grew up to 83%. These data indicate that the empty cloning vector immunization had no clinical effect on the immune system, whereas the parasitic infection did (Table 2).

All dogs inoculated with SS and later experimentally infected had lymph node enlargement only during t3, suggesting that the parasite stimulates the immune system, which manifests as lymphadenomegaly (Table 2).

### Effect of plasmid DNA immunization on heart rate

**Figure 3:**
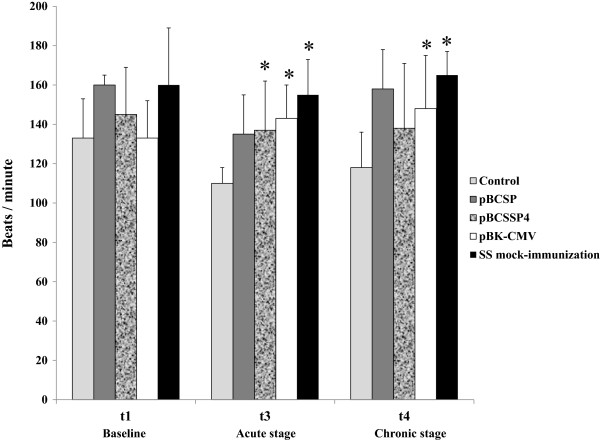
**Heart rate of DNA-immunized dogs with experimental *T. cruzi *infection.** Heart beat recordings from each experimental group (plasmid DNA immunized- and SS mock-immunized dogs) were compared with those of the control group (uninfected healthy animals). Heart rates exceeding 160 beats per minute were considered tachycardic. The values represent the average of the heart rate recordings ± S.D. Two-way ANOVA statistical test (* *P* ≤ 0.05).

The pBCSP plasmid-immunized/infected group did not have an increase in heart rate post-infection (t3 or t4) (Figure 3). The pBCSSP4 plasmid-immunized/infected group had a significant increase in heart rate during t3 but did not reach the 160 beats per minute that would indicate tachycardia; at t4 one dog had tachycardia, representing no statistical significance (Figure 3). Immunization with the pBK-CMV plasmid was unable to inhibit heart rate increases at any stage of the infection, showing a significant increase in pulse rate during both t3 and t4, as well as mock-immunized dogs with SS (Figure 3). These results indicate that immunization with the pBCSSP4 plasmid had a protective effect only during the chronic stage of the experimental infection; whereas immunization with the pBCSP plasmid had a greater protective effect since there were no increases in heart rate during both stages of CD.

### Effect of plasmid DNA immunization on heart electrical conduction during CD

Before immunization and challenge, 23% of the animals had sinus tachycardia, which was likely due to a physiological state associated with the restraint procedures used during the electrocardiographic recordings.

Seventeen percent of the dogs belonging to the pBCSP-immunized/infected group at t3 (Table 3) showed an increased duration of the P wave (0.06 s vs. 0.04 s), the absence of T waves in some records, and tachycardia. A P-R interval longer than the normal 0.12 s (0.14 s) was observed in 17% of the animals of this same group at the acute stage of CD. The conditions associated with these findings are intraventricular conduction defects, such as right or left bundle branch block (BBB) or ventricular enlargement, as well as an atrioventricular (AV) block. At t4 in this group, patterns characteristic of left ventricular enlargement, such as an abnormal left electrical axis deviation, were observed. Additionally, the R wave exceeded 2.5 mV in CV_6_LL (3.0 mV), and the R wave in lead I was greater than in lead III and aVF in 17% of the dogs.

**Table 3:** **Abnormal electrocardiographic features in DNA-immunized dogs with experimental *T. cruzi *infection**

**GROUP**	**3 MONTHS PI (t3 = ACUTE STAGE)**	**SUGGESTED PATHOLOGICAL CONDITIONS [**[23]**,**[24]**]**	**DOGS/n (%)**	**6 MONTHS PI (t4 = CHRONIC STAGE)**	**SUGGESTED PATHOLOGICAL CONDITIONS [**[23]**,**[24]**]**	**DOGS/n (%)**
**pBCSP**	Long P wave (0.06 s vs. 0.04 s ^a^), absence of some T waves, and tachycardia	Intraventricular conduction defects	1/6 (17%)	MEAD to the left, tall R wave in CV_6_LL (3.0 mV vs. 2.5 mV^a^), R wave greater in lead I than leads III and aVF	Left ventricle enlargement	1/6 (17%)
	Long P-R interval (0.14 s vs. 0.12 s ^a^)	AV block	1/6 (17%)			
**pBCSSP4**	Elevated S-T segment in leads I, III, aVF and CV_6_LL (0.5 mV vs. 0.2 mV ^a^)	Myocardial infarction and/or pericarditis	1/6 (17%)	Several absent P waves	Second-degree AV block	1/6 (17%)
				Wide QRS complex (0.06 s vs. 0.05 s^a^), inverted QRS complex in leads aVR, aVL, and CV_5_RL, and a small Q in lead I (0.05 mV)	Left BBB	1/6 (17%)
**pBK-CMV**	Reversal polarity of the T wave, R wave greater than 2.5 mV in lead II, III, aVF, and CV6LL, MEAD less than +40º	Left ventricular enlargement	3/6 (50%)	Reversal polarity of the T wave	Most often abnormal if it is found on serial EKG	2/6 (33%)
				+ T wave more than 25% larger than the R wave, QRS complex with S wave in leads I, II, III, aVF, CV_6_LL and CV_6_LU	Right BBB	1/6 (17%)
				MEAD to the left, R wave exceeded of 2.5 mV in lead CV_6_LL, R wave in lead I greater than in leads III and aVF	Left ventricle enlargement	3/6 (50%)
**SS (mock-immunized)**	MEAD to the left, R wave exceeded of 2.5 mV in lead CV_6_LL, R wave in lead I greater than in leads III and aVF	Left ventricle enlargement	2/6 (33%)	MEAD to the left, R wave exceeded of 2.5 mV in lead CV_6_LL, R wave in lead I greater than in leads III and aVF	Left ventricle enlargement	3/6 (50%)
	+ arrhythmia	Intraventricular conduction defects	1/6 (17%)	Rhythm irregular, QRS complex premature, bizarre, and of large amplitude, T wave directed opposite the QRS	VPC	1/6 (17%)
	+ prolonged Q-T interval	Intraventricular conduction defects	1/6 (17%)	Reversal in polarity of the T wave	Most often abnormal if it is found on serial EKG	2/6 (33%)

*PI* post-infection.

^a^ Reference values [23,24].

*BBB* bundle branch block.

*AV* atrioventricular.

*MEAD* mean electrical axis deviation.

*VPC* ventricular premature complexes.

An S-T segment elevation of 0.5 mV in leads II, III, aVF and CV_6_LL was observed in 17% of the dogs at t3 in the pBCSSP4-immunized/infected group (Table 3), which could suggest myocardial infarction and/or pericarditis. At t4, several P waves were not followed by QRS-T complexes in one of the dogs in this group, suggesting a second-degree AV block. At this time, the same dog that showed a possible myocardial infarction and/or pericarditis at t3 showed a wide QRS complex (0.06 s vs. 0.05 s), an inverted QRS complex in leads aVR, aVL and CV_5_RL, and a small Q in lead I (0.05 mV) indicative of left BBB, which might be caused by a myocardial disease such as the dilated form of chronic Chagas cardiomyopathy.

Reversal in polarity of the T wave combined with an R wave greater than 2.5 mV in leads II, III, aVF, and CV_6_LL and a mean electrical axis deviation (MEAD) in the frontal plane of less than +40° were found in 50% of the animals from the pBK-CMV-immunized/infected group (Table 3) at t3. These features suggested left ventricular enlargement that could be associated with a dilated form of cardiomyopathy caused by CD. Reversal in polarity of the T wave in 33% of the animals occurred again at the chronic stage of CD (t4), of which one dog (17%) showed a T wave that was increased more than 25% than the R wave, and QRS complexes had large wide S waves in leads I, II, III, aVF, CV_6_LL, and CV_6_LU that suggested right BBB.

Approximately 33% of the dogs from the SS-inoculated/infected group (Table 3) during t3 showed a MEAD to the left of the frontal plane combined with an R wave that exceeded 2.5 mV in lead CV_6_LL and an R wave in lead I greater than in leads III and aVF, suggesting left ventricle enlargement; two of these animals also had arrhythmia and a prolonged Q-T interval that suggested intraventricular conduction defects. The features associated with left ventricle enlargement such as a MEAD to the left of the frontal plane combined with an R wave above 2.5 mV in lead CV_6_LL and an R wave in lead I greater than in leads III and aVF appeared in 50% of the dogs during t4 in this group. Electrocardiographic features associated with ventricular premature complexes (VPC), such as rhythm irregularity, QRS complex premature, bizarre, and of large amplitude, and a T wave directed opposite the QRS, were also present in 17% of the dogs; reversal in polarity of the T wave in 33% of the animals occurred at this time (t4).

The control group animals were not subjected to any experimental procedure and exhibited normal electrocardiogram (EKG) at all times.

## Discussion

The results obtained in this study show classic clinical signs of CD described by others [6,15,25,26]. The sample size was appropriate for the DNA vaccination canine model in CD and was similar to what has been reported by other research groups in Mexico and other countries of America [6,8,26-29].

The DNA-immunized/infected dogs did not have body temperature increases during the acute phase of the disease. The hypothesis of glandular hypertrophy in the murine model during the acute stage of CD has been reported, mainly because of numerous parasite nests in the gland parenchyma with atrophy of secretory structures [30]. This event could have taken place in our model; however, we were unable to confirm this since no histological analysis during this phase of the present study was performed. The dogs immunized with the recombinant plasmids had clinically evident immune responses as evidenced by lymph node enlargement, however, during the post-infection period, the lymphadenopathy was less noticeable. To mount an immune response, lymphocytes must re-circulate between the blood and lymph nodes, recognize antigens upon contact with specialized presenting cells, proliferate to expand a small number of clonally-relevant lymphocytes, differentiate to antibody-producing plasma cells or effector T cells, exit from lymph nodes, migrate to tissues, and engage in host-protective activities such as lymphadenomegaly or fever [31]. Alternatively, fever has not been established as a constant sign of CD in dogs, but other physical signs such as lymph node enlargement always occur [13-15].

In dogs, the tachyarrhythmias have been documented in a continuous or paroxistic (intermittent) manner and are associated with several etiologies, including dilated myocardiopathy, which can be caused by CD, hypertrophic myocardiopathy, endocarditis, valvular disease, congenital defects, cardiac tumors, etc. These may worsen and progress to heart failure, which may result in death [32]. In our study, immunization with the recombinant plasmids was able to halt the heart rate increase during the acute and/or chronic stages of CD, which diminished tachyarrhythmia presentation.

Electrocardiographic tracings were analyzed according to the published data in canine and feline cardiology [23,24] and our results can be compared to human patients with CD [33-36]. Some electrocardiographic findings observed in this study are consistent with the most frequent electrocardiographic features reported when the canine model has been used in CD studies [6,8,15,25,26], such as myocardial infarction and/or pericarditis, second-degree AV block, left ventricle enlargement (dilation or hypertrophy), left and right BBB, and VPC.

In disagreement with findings reported by others [8,9,26,28], we did not observe any severe life-threatening arrhythmias, which is likely due to the strain used. Any dog infected with the *T. cruzi* Ninoa strain exhibited clinical signs that were severe enough to result in death. We chose the Ninoa strain as a reference Mexican strain, whose characterization fulfilled the criteria for being classified as biodeme 3 because of its biological behavior, tissue tropism, and virulence in a susceptible model [22]. Here, we demonstrate that the Ninoa strain of *T. cruzi* is able to establish an infection with the development of some characteristic clinical signs of acute and chronic stages of CD as described in dogs [13-15].

In a previous study with mice immunized with the pBCSP and pBCSSP4 plasmids, the *T. cruzi TcSP* and *TcSSP4* genes were also effective at inducing antigen-specific antibodies that recognized the surface of *T. cruzi* and produced a Th1 immune response, which conferred significant protection against *T. cruzi* experimental infection in BALB/c mice [17,18]. Our results indicate that the protective effect provided by the *TcSP* and *TcSSP4* genes was similar; however, immunization with the pBCSP plasmid avoided heart rate increases in both stages of the infection and prevented severe heart conduction abnormalities such as myocardial infarction, second-degree AV block or left BBB. This plasmid encodes the trans-sialidase superfamily protein that is present in all three forms of *T. cruzi* and is recognized by sera from chagasic patients, unlike the *TcSSP4* gene that codes for a glycoprotein associated with the transformation of trypomastigotes into amastigotes [37]. Thus, the slight difference in the protective effect is likely because the *TcSP* gene induces immunity against all forms of the parasite, whereas the *TcSSP4* gene only does so against a stage-specific surface antigen present on the amastigote form. Further studies of immunizations with the two plasmids in combination are required to determine if there is a possible synergistic effect.

We conclude that plasmid DNA vaccination with *T. cruzi* genes induces a moderate level of protection in immunized dogs because this strategy 1) avoids acute phase fever, 2) induces an immune response that manifests as lymph node enlargement as part of host-protective activity, 3) avoids heart rate increases during the acute and/or chronic stages, and 4) most interestingly, halts the symptomatic progression to severe heart conduction abnormalities.

## Abbreviations

CD: Chagas disease; pBCSSP4: plasmid containing the *TcSSP4* gene; pBCSP: plasmid containing the *TcSP* gene; ELISA: Enzyme-linked immunosorbent assay ELISA; *TcSSP4*: Gene encoding an amastigote-specific surface protein in *T. cruzi*; *TcSP*: Gene encoding a member of the trans-sialidase family in *T. cruzi*; pBk-CMV: Commercial empty plasmid used as a cloning vector; SS: Saline solution; IIF: Indirect immunofluorescence; ANOVA: Analysis of variance; BBB: Bundle branch block; AV: Atrioventricular; MEAD: Mean electrical axis deviation; VPC: Ventricular premature complexes; EKG: Electrocardiogram.

## Competing interests

Sources of financial support have been acknowledged and the authors declare that they have no competing interests.

## Authors' contributions

ORM and MAF participated in the concept and design of the study, conducted animal trials, collected and analyzed data, and completed manuscript preparation; MMPL carried out the EKGs and physical exams; MABV carried out the immunoassays; SCCS performed plasmid purification; RAA and MABV participated in metacyclic trypomastigote collection from bugs; JLRE, RAA and PAR contributed to the study design and assisted in drafting the manuscript. All authors read and approved the final manuscript.
